# Neuropsychiatric Features of a Cohort of Patients with Systemic Lupus Erythematosus

**DOI:** 10.5402/2012/989218

**Published:** 2012-11-20

**Authors:** Maria Francisca Moraes-Fontes, Isabel Lúcio, Céu Santos, Maria Manuel Campos, Nuno Riso, Manuel Vaz Riscado

**Affiliations:** ^1^Unidade de Doenças Auto-Imunes, Serviço 2-Medicina Interna, Hospital Curry Cabral, Centro Hospitalar Lisboa Central, Portugal; ^2^Neurorradiologia, Área de Diagnóstico por Imagem, Hospital de São José, Centro Hospitalar Lisboa Central, Portugal; ^3^Laboratório de Imunologia, Serviço de Imuno-Hemoterapia, Hospital Curry Cabral, Centro Hospitalar Lisboa Central, Portugal; ^4^Laboratório de Hemostase, Serviço de Imuno-Hemoterapia, Hospital Curry Cabral, Centro Hospitalar Lisboa Central, Portugal

## Abstract

In order to establish if neuropsychiatric systemic lupus erythematosus (NPSLE) can be identified by any characteristic other than those used to diagnose the neuropsychiatric (NP) disease itself, we retrospectively reviewed 98 systemic lupus erythematosus (SLE) patients followed over a mean period of 10 years. NPSLE was identified in 22 patients. Stroke and generalized seizures were the most frequent NP manifestations. The NPSLE and non-NPSLE groups were similar with regard to demographic characteristics, ACR criteria, serum autoantibodies, and frequency of hypertension and hypercholesterolemia. Of note, compared to the non-NPSLE group, NPSLE was associated with a higher frequency of smoking (78 versus 26%), organ damage (73 versus 34%), and cumulative mortality rate (14 versus 7%). The series of patients was further analysed according to the presence of antiphospholipid syndrome (APS). Significantly, the interval between the onset of NP disease and SLE diagnosis was shorter in the APS^−^ (0.3 ± 1 years) than in the APS^+^ (5 ± 7 years) groups. Recurrence and/or persistence of NP events were only documented in the APS^−^ group. Overall cumulative mortality was highest in NPSLE and in APS^+^ patients with inadequate anticoagulation control, identifying an aspect that requires improved vigilance and the development of novel therapeutic modalities.

## 1. Introduction

 In the course of their disease, many patients with systemic lupus erythematosus (SLE) develop neurologic and psychiatric symptoms. According to recent reviews neuropsychiatric (NP) disease occurs in as many as 30–56% of all SLE patients [1, 2]. However, the diagnosis of neuropsychiatric SLE (NPSLE) remains difficult. In a prospective study, only approximately one-fourth of NP events were attributed to SLE [3]. In addition, the proportion of NP cases amongst SLE patients may be overestimated because events such as cognitive impairment, mood, anxiety disorders, and headaches depend on assessing the subjective complaints of patients and are very frequent in the general population. The American College of Rheumatology (ACR) has listed 19 clinical entities that define NPSLE [4], but these do not differentiate, in population-based studies, NPSLE patients from non-SLE controls. The exclusion of headache, mild mood disorders, anxiety, mild cognitive dysfunction, and polyneuropathy without electrophysiological confirmation decreases the frequency of NPSLE diagnosis by half and increases the specificity of the ACR criteria from 46% to 93% [5]. NP events attributed to SLE occur mainly in the 6 months prior to, and in the first year following the diagnosis of SLE. However, these may be observed as late as 15 years after the initial diagnosis of SLE [6]. Although the life expectancy of patients with SLE has significantly improved over the past 50 years [7–10], NPSLE patients have a poorer quality of life than non-NPSLE patients [3, 6].

 There are no unequivocal clinical parameters or definitive laboratory tests for the diagnosis of NPSLE. Anti-Sm [11], antiribosomal P [12], and anti-N-methyl-D-aspartate receptor subunit antibodies [13–16] are associated but are not specific for NPSLE. The recognition of the antiphospholipid syndrome (APS) is critical for the institution of appropriate therapy [1, 17], but it occurs in SLE patients with or without NP disease. Neurological symptoms are significant manifestations of APS, including the recently described reversible posterior leucoencephalopathy syndrome [18], and endothelial dysfunction is well documented in SLE and APS [19]. Computed tomography (CT) does not recognize the diffuse presentations that may be detected by brain magnetic resonance imaging in the brains of NPSLE patients [20, 21]. However, in the absence of other diagnostic criteria the usefulness of MRI for the diagnosis of NPSLE is limited, since the lesions it detects are observed in healthy individuals [22] and in many SLE patients with no NP symptoms [23, 24]. MRI is the most useful for detecting and monitoring vascular ischemic and demyelinating lesions. In the absence of a diagnostic gold standard for NPSLE, it is essential to exclude other possible causes of NP symptoms such as infections, or metabolic disturbancies using a combination of CSF analysis, imaging, and electroencephalography [1].

 With the objective of establishing and defining characteristics of NPSLE patients we have conducted a retrospective analysis of a series of SLE patients currently attending an Autoimmune Disease (AID) Unit. We describe the identification of NPSLE patients with and without APS, followed by their comparison to SLE patients with no NP disease (non-NPSLE) taking into account demographic, laboratory, and imaging features as well as organ damage and survival. 

## 2. Patients and Methods

### 2.1. Patients

 The Autoimmune Disease Unit (AID) at the Hospital Curry Cabral in Lisbon, Portugal, is a referral centre for SLE patients. Since the establishment of the unit in 1993, the diagnosis of SLE was made in 163 patients of whom 11 died, 38 were lost to follow-up, and 43 were transferred to other units (known to be alive). The present study includes 98 patients under current follow-up. All fulfil the 1997 revised American College of Rheumatology (ACR) criteria for the diagnosis of SLE [25]. The date at which the fourth SLE classification criterion has been observed is recorded as the date of diagnosis. A patient was retrospectively identified as having NPSLE based on clinical diagnosis, according to the 1999 ACR-defined clinical entities and in the absence of an underlying non-SLE disease [4]. The date of the initial NP event was taken as date of diagnosis, and subsequent and persistent NP events were recorded. None of the patients are considered to have NPSLE had evidence of infections or severe electrolyte imbalance. NP symptoms as side effects of drugs were also excluded. Smoking, systemic arterial hypertension, and hypercholesterolemia were recorded at the time of the first NP or nephritis event. All patients were clinically evaluated at least twice per year, some as frequently as every month. Details of the medical history and examination, haematological, biochemical, and serologic variables related to the assessment of SLE were analysed. Patients considered to have APS fulfilled the Sydney classification criteria [26], with at least one major clinical criteria (vascular thrombosis and/or pregnancy morbidity) and tested positive for lupus anticoagulant (LAC), anticardiolipin antibodies (ACA), and anti-*β*2 glycoprotein I (*β*2-GPI) at least twice on two separate occasions. 

### 2.2. Laboratory Tests

 Serologic tests were performed at the Immunology Laboratory of Hospital Curry Cabral. Antinuclear antibodies (ANA) were detected by indirect immunofluorescence (IIF) using HEp-2 epithelial cells as the substrate (American Type Culture Collection CCL 23). The serum dilution was 1/160, and a titre equal to or greater than 1 : 160 (20 IU) was considered positive. The specificity of the ANA, namely, SSA, Sm, ribonucleoprotein (U1RNP), histone, nucleosome and ribosomal P proteins was determined by immunoblot line assay. Serum samples were diluted 1/10 for detection of antibodies against double stranded DNA on *Crithidia Luciliae*. Positive results were quantified by enzyme-linked immunosorbent assay (ELISA) for IgG. ELISA assays were also used for the quantification of IgM and IgG antibodies against anti-cardiolipin and anti-*β*2-GPI antibodies, and patients with antibodies (>20 MPL or GPL units) on two occasions, at least 12 weeks apart, were considered to be positive. To test for the lupus anticoagulant, phospholipid-dependent assays were performed at the Haemostasis Laboratory of Hospital Curry Cabral. The diluted Russell viper venom time (LAC Screen/LAC Confirm) was used together with Silica clotting time (SCT) (with low and high concentration of phospholipids). Prothrombin-time tests were performed to monitor warfarin therapy, and the results were expressed as international normalized ratios. Cerebrospinal fluid (CSF) was analysed for cells, protein, oligoclonal banding, Ziehl-Nielsen stain, and bacterial/mycobacterial culture. PCR for JC virus was only performed since 2010.

### 2.3. Neuroimaging and Vascular Assessment

 Assessment of the neurological system included cerebral CT scan and MRI, electroencephalography, nerve conduction studies, and lumbar puncture (LP). The exams were performed as judged appropriate by the attending physician according to the neuropsychiatric deficit, on a case by case basis. In all patients with strokes, echocardiograms and carotid Doppler ultrasound allowed for exclusion of atheroembolic disease.

### 2.4. Scores

 Global disease activity was quantified by the SLE Disease Activity Index (SLEDAI) [27] and cumulative organ damage through the Systemic Lupus International Collaborating Clinics (SLICC)/ACR Damage Index (SDI) [28]. The presence of organ damage was considered when the SDI score was ≥1. Retrospective determination of both scores has been validated [29, 30]. 

### 2.5. Statistics

 Descriptive statistics were used for all variables with percentages and mean ± standard deviation (SD). The Student' *t* and Mann-Whitney *U* tests were used for analysing quantitative and conventional chi-square and Fisher for qualitative differences. A *P* value <0.05 was taken to indicate statistical significance. 

## 3. Results

### 3.1. SLE Patient Classification

 Ninety eight patients currently followed for SLE at the AID Unit of the Curry Cabral Hospital in Lisbon were diagnosed between 1993 and 2010 and followed up for a mean duration of 9 ± 5 years. The patients were divided into two major groups, namely, patients with and without SLE-related NP disease, according to the presence of 1999 ACR-defined neurological clinical entities [4]. The two groups are referred to as NPSLE and non-NPSLE. Both groups were further divided into patients with and without the antiphospholipid syndrome, referred to as APS^+^ and APS^−^, respectively. Anti-cardiolipin, anti-*β*2-GPI, or LAC was present in 43% of all SLE patients, and the frequency was similar in NPSLE and non-NPSLE. After exclusion of 3 patients in whom cerebral events were the defined thrombotic phenomena, APS occurred in a similar percentage of NPSLE and non-NPSLE patients (27 versus 26%). The non-NPSLE group was further divided into patients with and without nephritis, and in each of these subgroups were APS^+^ and APS^−^ cases. Defining characteristics of patients in all subgroups are shown in Supplementary Data 1 (see Supplementary Material available online at doi:10.5402/2012/989218). 

### 3.2. Identification of Patients with NPSLE Disease

 NPSLE disease was identified in 22 of the 98 SLE patients. The clinical, brain MRI, and EMG findings that contributed to the diagnosis of NPSLE are shown separately for APS^−^ and APS^+^ NPSLE patients in Tables 1(a) and 1(b), respectively. NP manifestations included (in descending order of frequency) cerebrovascular disease (7/22; 38%), generalized seizures (6/22; 27%), major depression (4/22; 18%), severe headache (4/22; 18%), peripheral neuropathy (4/22; 18%), and in single patients (8%) severe cognitive dysfunction, acute confusional state, and Guillain-Barré syndrome. Isolated mild symptoms of headaches, anxiety, depression, or cognitive impairment were not considered as SLE-associated NP disease. Active lupus nephritis was present in three patients but neither in these patients with kidney disease, nor in other NPSLE patients were there evidences of other possible causes of NP disease such as side effects of drugs, severe electrolyte imbalance, or uremia. One patient was on regular and efficient dialysis for five years when peripheral neuropathy was diagnosed. Neuroimaging demonstrated a cerebral infarct in those patients with a clinical diagnosis of isolated ischemic stroke. Multiple focal lesions in the deep white matter, hyper-intense in T2-weighted images, and FLAIR were present in some patients with generalized seizures and depression. In retrospect, since lumbar puncture was only performed in five patients, infections of the brain could not be rigorously excluded for most of the NPSLE patients at the time of the NP event. 

### 3.3. Comparison of APS^+^ and APS^−^  NPSLE Patients

 The two groups were very similar except for two striking differences. First, all six patients with generalized seizures were in the APS^−^ groups. Second, the interval between the initial NP symptoms and the diagnosis of SLE was short (0.3 ± 1 year) for APS^−^ and much longer (6 ± 7 years) for APS^+^ patients. Strokes, peripheral neuropathy, and the simultaneous occurrence of more than one initial NP event were observed with a similar frequency in APS^+^ and APS^−^ groups. There were no obvious differences between the APS^+^ and APS^−^ groups with respect to demographic characteristics, the number and type of ACR criteria (Supplementary Data 1), and cumulative presence of autoantibodies in the serum (Supplementary Data 2). Recurrent and persistent NP manifestations such as severe headache, depression, and/or confusion were exclusively seen in four patients in the APS^−^ group. Two of these patients with recurrent NP symptoms also had recurrent lupus nephritis and another had significant *de novo* proteinuria (Supplementary Data 3). In these patients the findings in repeated MRI scans remained unchanged. Details of therapy are provided in Supplementary data 1 and 3.

### 3.4. Comparison of NPSLE and Non-NPSLE Patients

 NPSLE and non-NPSLE patients were compared with regard to demographic characteristics (gender, age, ethnicity, country of origin, marital status, progeny, and postsecondary education), duration of disease and follow-up, ACR criteria, laboratory findings, lupus disease activity, risk factors for cardiovascular disease, organ damage, and survival. 

#### 3.4.1. Demographic Characteristics, Disease Duration, and Follow-up 

 The demographic characteristics were very similar in the NPSLE and non-NPSLE groups (Table 2). Patients were predominantly women, Caucasian, born in Portugal, had a similar mean age of disease onset (44 ± 12 and 45 ± 14 years), an almost identical disease duration (13 ± 7 and 13 ± 8 years), and mean follow-up (10 ± 5 and 9 ± 5 years), respectively. 

#### 3.4.2. ACR Criteria and Autoantibodies Not Included in the ACR Criteria

 The NPSLE and non-NPSLE groups were very similar with regard to the number of ACR criteria as well as the type of ACR criteria (Table 3). The most frequent ACR criteria in both groups were arthritis, malar rash, photosensitivity, antinuclear, anti-dsDNA, and antiphospholipid antibodies. Antibodies against SSA, ribonucleoprotein, and histone were observed with similar frequencies in both patient groups (data not shown). In particular, antiribosomal P antibodies do not distinguish between both groups (the frequencies in NPSLE and non-NPSLE were 24 and 16% respectively, and this difference is not statistically significant). 

#### 3.4.3. Lupus Disease Activity

 In order to compare SLEDAI scores between NPSLE (at the time of the inaugural NP event) and non-NPSLE patients, we chose the group of non-NPSLE nephritis patients for whom it was possible to retrospectively calculate the score, at the time of nephritis presentation. The mean SLEDAI values were 15 ± 10 in NPSLE and 10 ± 4 in the isolated nephritis group. However, when the SLEDAI values were calculated for the NPSLE group without the NP component scores, the mean value became 6 ± 4 indicating low generalized disease activity in this group (data not shown). 

#### 3.4.4. Cardiovascular Risk Factors

 The frequency of patients who smoked was determined at the time of the defining NP event, nephritis diagnosis or SLE diagnosis in the non-NPSLE, nonnephritis patients. Smokers were significantly more frequent in the NPSLE group (78%, *n* = 15) when compared to non-NPSLE (26%, *n* = 20), and this difference were statistically significant (*P* = 0,001). Hypertension and hypercholesterolemia was present in the lupus nephritis group at a frequency of 65% each. These risk factors were found at a similar frequency in the NPSLE (23 and 22%, resp.) and non-NPSLE nonnephritis groups (28 and 22%).

#### 3.4.5. Organ Damage

**Figure 1:**
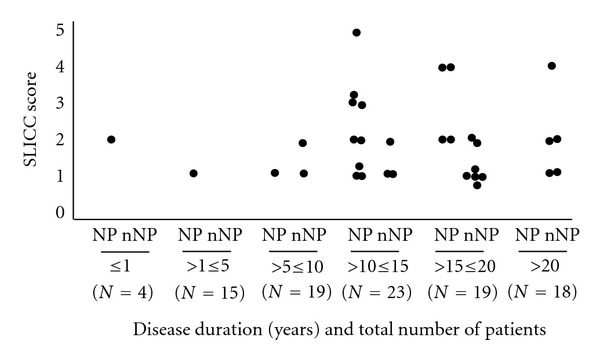
Final SLICC scores according to duration of follow-up in NPSLE (NP) and non-NPSLE (nNP) patients.

 Pathologies known to be caused by the treatment with steroids (such as osteonecrosis, osteoporotic fractures, cataracts, and hyperglycaemia) and neoplasia occurred in both groups. Cumulative organ damage was quantified as the Systemic Lupus International Collaboration Clinics/ACR Damage Index (SDI). Organ damage as indicated by SDI values ≥ 1 was observed in 73% of NPSLE and in 34% of non-NPSLE patients (Table 4). This difference is statistically significant (*P* = 0,0001) and largely due to the damage of the neurologic system that is evident in 14 of the 22 NPSLE patients, but absent in all non-NPSLE patients. The difference between final and initial SDI scores was, however, not significantly different between the two groups. Scores increased with the duration of the disease in both groups and tended to be higher in NPSLE patients (Figure 1). 

#### 3.4.6. Mortality

**Figure 2:**
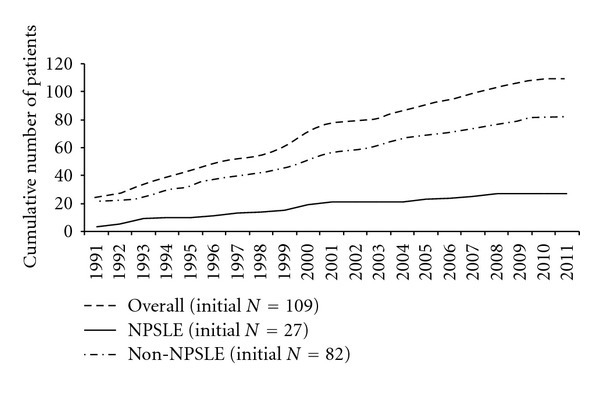
Cumulative mortality shown for all SLE patients and for each patient group (NPSLE and non-NPSLE). During follow-up, 5 NPSLE patients and 6 non-NPSLE patients died.

 The cumulative mortality is shown in Figure 2 for all SLE patients as well as for the NP and non-NPSLE groups, and the causes of death are shown in detail in Supplementary Data 4. Mortality was highest in the NPSLE group, but the difference to the non-NPSLE group was not statistically significant. Eleven patients died over an eighteen-year follow-up. The average disease duration of the patients who died was 13 ± 5 years. Six of the patients who died had NPSLE, of which 4 fulfilled the criteria for APS. The 6 patients who were positive for antiphospholipid antibodies died at a younger age (40 ± 13 versus 64 ± 8) (*P* = 0,04). Of note, 3 patients had an infratherapeutic INR at the time of the thrombotic event that caused death, only one patient died of an infection, and one patient died of cancer. None of the patients died from renal failure. 

## 4. Discussion and Conclusions

 The objective of this work was to find features beyond those associated with the clinical diagnosis of NPSLE that may characterize this subgroup of patients. We report a detailed description of 22 patients with NP manifestations corresponding to 23% of our SLE patients under current follow-up. This frequency is within the range of 12 to 30% previously reported in Portuguese series [31, 32]. In addition, this group of patients was not different from large, previously studied cohorts with regard to cumulative clinical manifestations [33–36], frequency of anti-cardiolipin antibodies [37, 38], and APS [39]. We also failed to find significant differences between NPSLE and non-NPSLE with regard to demographic features, the age at which SLE was diagnosed, the disease duration, and follow-up time, in spite of previous reports that NPSLE is less frequent in Caucasians [3].

 There was no significant difference between the NPSLE and non-NPSLE groups in the frequency of skin lesions such as malar rash, discoid lesions, photosensitivity, and in the occurrence of isolated discoid lupus before SLE diagnosis. Once again, these findings are in accordance with previous observations in a prospective study of a large NPSLE cohort [40] and in several other studies [1]. Likewise, no tests for circulating antibodies were differentially found in our patients with NPSLE. In particular, we found antiribosomal P antibodies in both NPSLE (36%) and non-NPSLE patients (33%). The association between these antibodies and NP disease in SLE patient has been controversial [41–43], but a recent meta-analysis concluded that circulating antiribosomal P antibodies do not predict nor confirm the occurrence of NP disease in SLE patient [12]. 

 Smoking aggravates end stage renal disease in lupus nephritis [44], decreases the efficacy of antimalarial agents in cutaneous lupus [45], and contributes to arterial thrombotic events, in particular in patients with antiphospholipid antibodies [46]. Our finding that the proportion of smokers was significantly higher in patients with NPSLE suggests that smoking may also contribute to the development of the NP disease in SLE patients.

 Lupus disease activity was higher in the NPSLE group. However, when the activity scores related to the NP disease were excluded the SLEDAI was not significantly different in patients with and without NP disease. Generalized high SLE disease activity is considered to be a risk factor for NPSLE [1], but this may be overestimated by the fact that scoring systems attribute a high score to NP symptoms and signs. In accordance with a previous study [47] we did find more organ damage in the NPSLE group which was, as expected, due to neurological disease. 

 Significantly, we found that a heterogeneous group of APS^−^ NPSLE patients could be distinguished from the APS positive group by the duration of SLE disease that preceded the NP event. According to the original ACR criteria, NP manifestations can precede the onset of lupus or occur at any time during its course, in both active SLE and quiescent periods, but a NP event is considered more likely to be SLE related if it has not preceded the diagnosis of SLE by more than 6 months [1, 3]. Ischemic stroke and generalized seizures were the most frequent NP manifestations in our patients as reported [1]. Unexpectedly, the frequency of ischemic stroke in our cohort was similar in the APS^+^ and APS^−^ groups. The APS^−^ NPSLE group was further characterized by seizures which were absent in the APS^+^ group. These were unusual findings and may be due to the small number of patients. Ischemic stroke occurs frequently in patients with the APS [48–51], and even though seizures are not part of the revised classification criteria for APS [26] they are well described in this entity [52, 53]. Recurrence and persistence of NP disease occurred exclusively in the APS-group and in three patients was associated with lupus nephritis. Cumulative mortality was higher in NPSLE patients, in particular in those with antiphospholipid antibodies and long disease duration. This finding is in accordance with the 10-year Eurolupus study, where cerebral thrombosis due to the APS was found to be the leading cause of death in SLE patients when death occurred after a time lapse of five years from the initial diagnosis [54].

 In summary, we have been unable to find differentiating traits that can be used in clinical practice between NPSLE and non-NPSLE patients. Within the NPSLE group of patients, the group of APS^−^ patients could be distinguished from the APS positive group by the fact that in the first group, the NP event occurred almost simultaneously with SLE diagnosis while in the latter, the event occurred at a much later time. Recurrence and persistence of NP disease were only observed in the APS-group of patients, frequently associated with the recurrence of nephritis. In addition, overall mortality was higher in NPSLE and APS patients with inadequate anticoagulation control, identifying an area that requires improved vigilance and the development of novel therapeutic modalities. The identification of a higher frequency of smokers in the NPSLE group deserves further understanding of the underlying pathogenetic mechanism and institution of preventative measures. 

## Supplementary Material

The following supplementary material includes a description of the group of Systemic Lupus Erythematosus (SLE) patients currently followed in The Autoimmune Disease Unit at the Hospital Curry Cabral in Lisbon, Portugal, at the time of writing.
Supplementary Data 1 provides demographic, clinical manifestations and therapeutic details, according to the presence of Neuropsychiatric Disease (NP), Nephritis and Antiphospholipid Syndrome (APS). Supplementary Data 2 compares cumulative autoantibody reactivities between the different groups of patients. Supplementary Data 3 describes the recurrence, persistence and therapy of Neuropsychiatric Symptoms in the Neuropsychiatric SLE (NPSLE) APS negative group of patients. Supplementary Data 4 enumerates the clinical characteristics of the patients that died in 18 years of follow-up.

## Figures and Tables

**Table:** (a)

ID	Age of the patient at the time of NP event (years)	Interval between NP event and SLE diagnosis (years)	Case definition	Brain CT scan/brain MRI/EMG
1*	16	−2	Generalized seizure disorder, acute psychosis, and severe hemicraneal headache	MRI: generalized cortical atrophy
2	11	0	Generalized seizure disorder	Not available
3*	30	0	Generalized seizure disorder	MRI: multiple focal deep white matter lesions hyperintense in T2-weighted images and FLAIR
4*	17	0	Generalized seizure disorder	MRI: no abnormality
5*	42	−1	Generalized seizure disorder, severe headache, major depression	MRI: subcortical high signal bifrontal, bilateral deep cortical, and corona radiata focal lesions in T2-weighted images.
6*	23	0	Generalized seizure disorder and ischemic stroke	MRI: right cerebral atrophy. Left thalamic calcified lesion.
7	49	0	Ischemic stroke	CT scan: ischemic infarct left hippocampus
8	31	1	Ischemic stroke	CT scan: left lenticular haemorrhagic infarct
9	50	2	Ischemic stroke	MRI: right ischemic parieto-temporal infarct
10	23	0	Peripheral neuropathy	EMG: motor-sensitive neuropathy
11	17	1	Peripheral neuropathy	EMG: motor-sensitive neuropathy
12*	45	0	Major depression, severe hemicraneal headache	MRI: multiple focal deep white matter high signal on T2-weighted images and FLAIR. Small focal lesions in the subcortical fronto parietal white matter, predominantly on the right.
13*	29	2	Major depression with suicidal ideation, cluster headache	MRI: multiple small focal subcortical white matter and right paramedian pontine high-signal lesions on T2-weighted images and FLAIR.

*Interictal EEG was performed in these patients. An epileptiform focus was detected in 3 (patients 3, 4, and 6) with no abnormality in the other 4 patients (patients 1, 5, 12, and 13). Nephritis was present in patients 3, 12, and 13 at the time of the NP event.

**Table:** (b)

ID	Age of the patient at the time of NP event (years)	Interval between NP event and SLE diagnosis (years)	ACR clinical entity	APS defining event	Brain CT scan/brain MRI/EMG
14	29	5	Ischemic stroke with hemorrhagic transformation	Cerebral thrombosis	MRI: subcortical right parietal ischemic infarct with hemorrhagic transformation
15	34	17	Ischemic stroke	Peripheral artery thrombosis	MRI: right parietal ischemic infarct
16	43	0	Ischemic stroke and 7 days later Guillain Barré Syndrome	Cerebral thrombosis	MRI: oval-shaped left subcortical paraventricular high-signal lesions on T2- and FLAIR-weighted images with a signal change in diffusion sequence and a left corona hemorrhagic infarct; EMG: motor-sensitive polyneuropathy
17	34	1	Peripheral neuropathy	Coronary thrombosis	EMG: motor-sensitive neuropathy
18	33	16	Peripheral neuropathy	Repeated dialysis access thrombosis	EMG: motor-sensitive neuropathy
19	38	−2	Major depression; ischemic optic neuropathy,	Retinal artery thrombosis	MRI: no abnormality
20*	43	14	Major depression	Limb deep vein thrombosis and obstetric loss	MRI: multiple focal subcortical lesions hyperintense in T2
21	28	0	Migrainous headaches and acute confusional state	Cerebral thrombosis	MRI: no abnormality
22*	35	14	Severe headaches and cognitive dysfunction	Obstetric loss	MRI: multiple focal deep white matter lesions hyperintense in T2 on T2-weighted images and FLAIR.

*Interictal EEG was performed in 2 patients (patients 20 and 22). Left temporal subclinical rhythmic electrographic discharges of adults—SREDA was detected in patient 22.

**Table 2:** Demographic characteristics, disease duration, and followup of NPSLE and non-NPSLE patients.

Characteristics	NPSLE *N* (%)	Non-NPSLE *N* (%)
Female	18 (82)	70 (92)
Male	4 (18)	6 (8)
Female : male ratio	4,5	11,7
Age, mean ± SD (y)	44 ± 12	45 ± 14
Caucasian	17 (77)	68 (89)
Non-Caucasian	5 (23)	8 (11)
Portugal	18 (82)	66 (87)
Portuguese speaking Africa	4 (18)	8 (11)
Brazil	0	2 (3)
Single	9 (41)	30 (39)
Nonsingle	13 (59)	46 (61)
Progeny	6 (27)	41 (54)
Postsecondary education	8 (36)	33 (43)
Disease duration, mean ± SD (years)	13 ± 7	13 ± 8
Followup, mean ± SD (years)	10 ± 5	9 ± 5

**Table 3:** ACR criteria in NPSLE and non-NPSLE patients.

ACR criteria	NPSLE *N* (%)	Non-NPSLE *N* (%)
Malar rash	14 (64)	29 (38)
Discoid rash	6 (27)	8 (11)
Photosensitivity	11 (50)	31 (41)
Oral ulcer	5 (23)	20 (26)
Arthritis	16 (73)	51 (67)
Serositis	2 (9)	11 (14)
Renal disorder	8 (36)	26 (34)
Haemolytic anemia	1 (5)	18 (24)
Lymphopenia	6 (27)	24 (32)
Thrombocytopenia	4 (18)	16 (21)
Antinuclear antibody	21 (95)	76 (100)
Anti-dsDNA antibodies	15 (68)	63 (83)
Anti-Sm antibodies	6 (27)	17 (22)
Anti-cardiolipin antibodies/anti-*β*2GPI/Lupus anticoagulant	9 (41)	33 (43)
Number of ACR criteria, mean ± SD	6 ± 2	5 ± 1

**Table 4:** Initial and final SLICC scores in NPSLE and non-NPSLE patients.

Characteristics	NPSLE	Non-NPSLE
Total number of patients	22	76
Total followup, mean ± SD	10 ± 5	9 ± 5
Number and % of patients with organ damage (SDI ≥ 1)	16 (73)	17 (34)
Initial SDI, mean ± SD	0,44 ± 0,89	0
Final SDI, mean ± SD	2,31 ± 1,25	1,53 ± 0,80
Delta SDI, mean ± SD	1,88 ± 1,15	1,53 ± 0,80
