# Supplementary material for: Neuropsychiatric Features of a Cohort of Patients with Systemic Lupus Erythematosus
Source: ISRN Rheumatol. 2012 Nov 20;2012:989218. doi: 10.5402/2012/989218 (PMC3512311; doi:10.5402/2012/989218)
Supplement: Supplementary file 1 — The following supplementary material includes a description of the group of Systemic Lupus Erythematosus (SLE) patients currently followed in The Autoimmune Disease Unit at the Hospital Curry Cabral in Lisbon, Portugal, at the time of writing. Supplementary Data 1 provides demographic, clinical manifestations and therapeutic details, according to the presence of Neuropsychiatric Disease (NP), Nephritis and Antiphospholipid Syndrome (APS). Supplementary Data 2 compares cumulative autoantibody reactivities between the different groups of patients. Supplementary Data 3 describes the recurrence, persistence and therapy of Neuropsychiatric Symptoms in the Neuropsychiatric SLE (NPSLE) APS negative group of patients. Supplementary Data 4 enumerates the clinical characteristics of the patients that died in 18 years of follow-up. [file 989218.f1.doc]

**Supplementary Data 1: Demographic and clinical manifestations of all currently followed-up SLE patients according to the presence of NP disease, nephritis and APS**

| **N (%)** | **All patients (N=98)** | **NPSLE (N=22)** | **Non-NPSLE (N=76)** | **NPSLE** | | **Non-NPSLE** | | | |
| --- | --- | --- | --- | --- | --- | --- | --- | --- | --- |
|  |  |  |  |  |  | **Nephritis (N=26)** | | **Non-NPSLE, non-nephritis (N=50)** | |
|  |  |  |  | **Non-APS (N=13)** | **APS (N=9)** | **Non-APS (N=22)** | **APS (N=4)** | **Non-APS (N=37)** | **APS (N=13)** |
| Gender: |  | | | | | | | | |
| Female | 88 (90) | 18 (82) | 70 (92) | 10 (77) | 8 (89) | 20 (91) | 3 (75) | 35 (95) | 12 (92) |
| Male | 10 (10) | 4 (18) | 6 (8) | 3 (23) | 1 (11) | 2 (9) | 1 (25) | 2 (5) | 1 (8) |
| Female:male ratio | 8,8 | 4,5 | 11,7 | 3,3 | 8 | 10 | 3 | 17.5 | 12 |
| Age, mean ± SD (y) | 44 ± 14 | 44 ± 12 | 45 ± 14 | 40 ± 13 | 44 ± 11 | 43 ± 12 | 36 ± 8 | 44 ± 15 | 55 ± 13 |
| Ethnicity: |  | | | | | | | | |
| Caucasian | 85 (87) | 17 (77) | 68 (89) | 12 (92) | 5 (56) | 21 (95) | 4 (100) | 31 (84) | 12 (92) |
| Non-Caucasian | 13 (13) | 5 (23) | 8 (11) | 1 (8) | 4 (44) | 1 (5) | 0 | 6 (16) | 1 (8) |
| Country of origin: |  | | | | | | | | |
| Portugal | 84 (86) | 18 (82) | 66 (87) | 12 (92) | 6 (67) | 19 (86) | 4 (100) | 31 (84) | 12 (92) |
| Portuguese Speaking African Countries | 12 (12) | 4 (18) | 8 (11) | 1 (8) | 3 (33) | 1 (5) | 0 | 6 (16) | 1 (8) |
| Brazil | 2 (2) | 0 | 2 (3) | 0 | 0 | 2 (9) | 0 | 0 | 0 |
| Marital Status: |  | | | | | | | | |
| Single | 39 (40) | 9 (41) | 30 (39) | 6 (46) | 3 (33) | 8 (36) | 2 (50) | 16 (43) | 4 (31) |
| Married | 50 (51) | 9 (41) | 41 (54) | 4 (31) | 5 (56) | 14 (64) | 2 (50) | 19 (51) | 6 (46) |
| Divorced | 6 (6) | 3 (14) | 3 (4) | 2 (15) | 1 (11) | 0 | 0 | 2 (5) | 1 (8) |
| Widow | 3 (3) | 1 (5) | 2 (3) | 1 (8) | 0 | 0 | 0 | 0 | 2 (15) |
| Progeny | 47 (48) | 6 (27) | 41 (54) | 3 (23) | 3 (33) | 10 (45) | 1 (25) | 22 (59) | 8 (62) |
| Post secondary education (>12 y school) | 41 (42) | 8 (36) | 33 (43) | 4 (31) | 4 (44) | 11 (50) | 1 (25) | 16 (43) | 5 (38) |
| Cumulative number of ACR criteria, mean ± SD | 5 ± 1 | 6 ± 2 | 5 ± 1 | 6 ± 1 | 6 ± 2 | 5 ± 1 | 6 ± 1 | 5 ± 1 | 5 ± 1 |
| Cumulative ACR manifestations: |  | | | | | | | | |
| Malar rash | 43 (44) | 14 (64) | 29 (38) | 9 (69) | 5 (56) | 8 (36) | 3 (75) | 14 (38) | 4 (31) |
| Discoid rash | 14 (14) | 6 (27) | 8 (11) | 4 (31) | 2 (22) | 0 | 0 | 5 (13) | 3 (23) |
| Photosensitivity | 45 (46) | 11 (50) | 31 (41) | 9 (69) | 2 (22) | 10 (45) | 0 | 16 (43) | 5 (38) |
| Oral Ulcer | 25 (26) | 5 (23) | 20 (26) | 2 (15) | 3 (33) | 4 (18) | 2 (50) | 12 (32) | 2 (15) |
| Arthritis | 67 (68) | 16 (73) | 51 (67) | 8 (62) | 8 (89) | 16 (73) | 0 | 27 (73) | 6 (46) |
| Serositis | 13 (13) | 2 (9) | 11 (14) | 0 | 2 (22) | 2 (9) | 4 (100) | 6 (16) | 3 (23) |
| Renal disorder | 34 (45) | 8 (36) | 26 (34) | 5 (38) | 3 (33) | 22 (100) | 0 | 0 | 0 |
| Neurologic disorder (according to 1999 ACR case definitions) | 22 (22) | 22 (100) | 0 | 13 (100) | 9 (100) | 0 | 0 | 0 | 0 |
| Haemolytic anemia | 19 (19) | 1 (5) | 18 (24) | 0 | 1 (11) | 5 (23) | 0 | 8 (22) | 5 (38) |
| Lymphopenia | 30 (31) | 6 (27) | 24 (32) | 3 (23) | 3 (33) | 6 (27) | 1 (25) | 12 (32) | 5 (38) |
| Thrombocytopenia | 20 (20) | 4 (18) | 16 (21) | 2 (15) | 2 (22) | 4 (18) | 2 (50) | 7 (19) | 3 (23) |
| Anti-nuclear antibody | 97 (99) | 21 (95) | 76 (100) | 12 (92) | 9 (100) | 22 (100) | 4 (100) | 37 (100) | 13 (100) |
| Anti-dsDNA antibodies | 78 (80) | 15 (68) | 63 (83) | 9 (69) | 6 (67) | 22 (100) | 3 (75) | 29 (78) | 9 (69) |
| Anti-Sm antibodies | 23 (23) | 6 (27) | 17 (22) | 2 (15) | 4 (44) | 4 (18) | 1 (25) | 11 (30) | 1 (8) |
| Anti-cardiolipin antibodies | 29 (31) | 6 (27) | 23 (32) | 0 | 6 (67) | 6 (29) | 3 (75) | 7 (21) | 7 (54) |
| Anti- β2PI | 25 (28) | 5 (23) | 20 (30) | 0 | 5 (56) | 6 (29) | 3 (75) | 5 (17) | 6 (50) |
| Lupus anticoagulant | 25 (28) | 6 (33) | 19 (29) | 0 | 6 (67) | 2 (10) | 4 (100) | 4 (13) | 9 (60) |
| Anti-phospholipid antibodies and/or Lupus anticoagulant | 42 (43) | 9 (41) | 33 (43) | 0 | 9 (100) | 6 (27) | 4 (100) | 10 (27) | 13 (100) |
| Cumulative Medication: |  | | | | | | | | |
| Steroids received at least once | 92 (94) | 22 (100) | 70 (92) | 13 (100) | 9 (100) | 22 (100) | 4 (100) | 33 (89) | 11 (85) |
| Long term maintenance (Mx) steroid therapy (≤ 6mg/d Pred equivalent) | 73 (74) | 18 (82) | 55 (72) | 10 (77) | 8 (89) | 17 (77) | 1 (25) | 29 (78) | 8 (62) |
| Long term maintenance (Mx) steroid therapy (> 6mg/d Pred equivalent) | 10 (10) | 4 (18) | 6 (8) | 3 (23) | 1 (11) | 3 (14) | 2 (50) | 1 (3) | 0 |
| Long term Mx steroids alone | 5 (5) | 0 | 5 (7) | 0 | 0 | 1 (5) | 0 | 3 (8) | 1 (8) |
| Long term Mx HCQ alone | 11 (11) | 0 | 11 (14) | 0 | 0 | 0 | 0 | 6 (16) | 5 (38) |
| Long term Mx steroids + HCQ only | 35 (36) | 11 (50) | 24 (32) | 6 (46) | 5 (56) | 5 (23) | 1 (25) | 15 (41) | 3 (23) |
| Long term Mx steroids + HCQ + AZA only | 34 (35) | 9 (41) | 25 (33) | 5 (38) | 4 (44) | 9 (41) | 2 (50) | 10 (27) | 4 (31) |
| Long term Mx HCQ + AZA only | 3 (3) | 0 | 3 (4) | 0 | 0 | 2 (9) | 1 (25) | 0 | 0 |
| MMF | 14 (14) | 5 (23) | 9 (12) | 3 (23) | 2 (22) | 7 (32) | 1 (25) | 0 | 1 (8) |
| Cyclophosphamide | 23 (23) | 10 (45) | 13 (17) | 5 (38) | 5 (56) | 10 (45) | 0 | 2 (5) | 1 (8) |
| Rituximab | 5 (5) | 4 (18) | 1 (1) | 4 (31) | 0 | 1 (5) | 0 | 0 | 0 |
| Age SLE diagnosis, mean ± SD (y) | 31 ± 13 | 29 ± 12 | 31 ± 14 | 30 ± 13 | 28 ± 10 | 29 ± 12 | 18 ± 6 | 31 ± 13 | 39 ± 16 |
| Follow-up, mean ± SD (y) | 9 ± 5 | 10 ± 5 | 9 ± 6 | 9 ± 6 | 11 ± 4 | 8 ± 4 | 9 ± 5 | 9 ± 6 | 9 ± 7 |
| Disease duration, mean ± SD (y) | 13 ± 8 | 13 ± 7 | 13 ± 8 | 11 ± 7 | 16 ± 6 | 14 ± 8 | 18 ± 12 | 13 ± 8 | 15 ± 9 |
| Time elapsed between SLE diagnosis and first NP or nephritis event (y) | - | 3 ± 6 | - | 0.3 ± 1.1 | 6 ± 7 | 6 ± 7 | 10 ± 10 | - | - |

The majority of patients received steroid therapy during the course of their disease and most patients were on maintenance long term low dose (< 6 mg/day of prednisolone or equivalent steroid formulation). HCQ is routinely prescribed but intolerance occurred in 15% of patients. More patients in the NPSLE group were treated during the course of disease with a steroid daily dose equivalent to > 6 mg of Prednisolone, none were managed with HCQ alone and more required combination immunosuppressive therapy including mycophenolate mophetil, cyclophosphamide and rituximab. In the APS**^+^** group, 3 patients received CYP (Patients 16, 20 and 22). There was diagnostic doubt in patients 20 and 22 whose MRI scan showed multiple focal hyperintense lesions and initial CYP was successfully replaced by warfarin once APS was diagnosed.

**Supplementary Data 2: Comparison of cumulative autoantibody reactivities between the different groups**

| **N (%)** | **All patients** | **NPSLE** | **Non-NPSLE** | **NPSLE** | | **Non-NPSLE** | | | |
| --- | --- | --- | --- | --- | --- | --- | --- | --- | --- |
|  |  |  |  |  |  | **Nephritis** | | **Non-NPSLE, non-nephritis** | |
|  |  |  |  | **Non-APS** | **APS** | **Non-APS** | **APS** | **Non-APS** | **APS** |
| Anti-nuclear antibody | 97 (99) | 21 (95) | 76 (100) | 12 (92) | 9 (100) | 22 (100) | 4 (100 | 37 (100) | 13 (100) |
| Anti-dsDNA antibodies | 78(80) | 15 (68) | 63 (83) | 9 (69) | 6 (67) | 22 (100) | 3 (75) | 29 (78) | 9 (69) |
| Anti-Sm antibodies | 23 (23) | 6 (27) | 17 (22) | 2 (15) | 4 (44) | 4 (18) | 1 (25) | 11 (30) | 1 (8) |
| Anti-histone | 41 (44) | 10 (45) | 31 (44) | 6 (46) | 4 (44) | 13 (65) | 3 (75) | 12 (32) | 3 (23) |
| Anti-nucleossome | 46 (55) | 12 (55) | 34 (54) | 6 (46) | 6 (67) | 11 (55) | 3 (75) | 15 (56) | 5 (45) |
| Anti-centromere | 0 | 0 | 0 | 0 | 0 | 0 | 0 | 0 | 0 |
| Anti-Scl-70 | 1 (1) | 1 (5) | 0 | 1 (8) | 0 | 0 | 0 | 0 | 0 |
| Anti-SSA | 44 (45) | 9 (41) | 35 (47) | 5 (38) | 4 (44) | 10 (48) | 1 (25) | 19 (51) | 5 (38) |
| Anti-ribo nucleoprotein | 32 (34) | 8(36) | 24 (33) | 2 (15) | 6 (67) | 5 (24) | 1 (25) | 15 (42) | 3 (23) |
| Anti-ribossomal | 17 (18) | 5 (24) | 12 (16) | 3 (23) | 2 (25) | 4 (19) | 1 (25) | 4 (11) | 3 (23) |

**Supplementary Data 3: Recurrence, persistence and therapy of Neuropsychiatric Symptoms in the NPSLE APS^-^ group of patients**

| **ID** | **Previous immunosuppressive therapy** | **Subsequent / persistent NP events attributed to NPSLE** | **Cumulative immunosuppressive therapy to which NPSLE was Refractory** | **Therapy for Refractory Disease** | **Effectiveness of Rituximab** |
| --- | --- | --- | --- | --- | --- |
| 3 | For nephritis: Multiple pulses Methylprednisolone, plasmapharesis and MMF failed, CYP (cumulative dose 6g- 8 monthly cycles) | Headache (cluster), anxiety disorder, cognitive dysfunction with recurrence of Class III nephritis (recurrence NP symptoms 10 years after first NP event) | Steroids, AZA, CyA, MMF, CYP | Yearly RTX x 3 | At 3 y follow-up:  Full remission nephritis, partial improvement of NP events |
| 5 | For neuropsychiatric disease: CYP (cumulative dose 9g - 12 monthly cycles), | Auditory hallucinations with probable nephritis - significant proteinuria, no biopsy (recurrence NP symptoms 10 years after first NP event) | Steroids, HCQ, AZA, CYP | RTX x 1 | At 1 y follow-up:  No proteinuria. no improvement in NP symptoms |
| 12 | For neuropsychiatric disease: MMF (1500 mg/d for 10 months) followed by CYP (cumulative dose 18 g- 24 monthly cycles) | Major depression - suicidal ideation with multiple psychiatric admissions (persistent NP symptoms) | Steroids, HCQ, AZA, MMF, CYP | Yearly RTX x 2 | At 2 y follow-up:  Partial improvement of NP events |
| 13 | For nephritis: MMF (2g/d for 6 months) followed by CYP (cumulative dose 5 g - 8 monthly cycles), | Cluster headache and major depression - suicidal ideation with recurrence of nephritis – Class IV (persistent NP symptoms) | Steroids, HCQ, AZA, MMF, CYP | Yearly RTX x 2 | At 2 y follow-up:  Full remission nephritis, partial improvement of NP events |

Four patients were considered to have cyclophosphamide refractory disease and were treated with rituximab. Amelioration of nephritis occurred in the three patients. Rituximab resulted in amelioration of headaches, cognitive dysfunction, depression and anxiety but there was no effect on the frequency of auditory hallucinations in patient 5.

**Supplementary Data 4: Clinical characteristics of the patients tha**t died in 18 years of follow-up*

| Age at time of death | Anticardiolipin Antibodies | Antiphos-pholipid Syndrome | Duration of Disease (y) | Number of ACR criteria | Previous Thrombosis | Previous nephritis | Number of years in nephritis remission | Case Definition NPSLE before death | Warfarin | Infra-theurapeutic INR | Cause of death |
| --- | --- | --- | --- | --- | --- | --- | --- | --- | --- | --- | --- |
| 41 | + | + | 8 | 6 | Cerebral | - | Not applicable (NA) | CVD - ischemic stroke | + | + | CVD - ischemic stroke |
| 63 | + | + | 5 | 4 | Cerebral | - | NA | CVD - ischemic stroke | - | Contraindicated due to large malignant pericardeal effusion | Malignant thymoma |
| 41 | + | + | 16 | 7 | Pulmonary embolus | - | NA | Mood disorder - major depression | + | + | Pulmonary embolus |
| 24 | + | + | 7 | 4 | Deep vein | - | 2 | CVD - ischemic stroke | + | + | Thrombotic stroke with hemorrhagic transformation |
| 36 | + | + | 17 | 4 | . | - | 7 | - | - | NA | Pulmonary embolus |
| 33 | + | - | 4 | 6 | - | + | 0 | - | + | NA | Septic shock from pneumonia |
| 69 | - | - | 17 | 6 | - | - | NA | - | NA | NA | Sudden, at home |
| 54 | - | - | 14 | 8 | - | + | 4 | - | NA | NA | Breast carcinoma |
| 68 | - | . | 17 | 4 | - | - | NA | Psychosis and cognitive dysfunction | NA | NA | Sudden, at home |

*Data only known for 9 of the 11 patients who died.
